# Talking About Physical “Activity” or “Inactivity”? The Need of Accurate Activity Controlling in Exercise Studies in Rodents

**DOI:** 10.3389/fphys.2020.611193

**Published:** 2020-12-08

**Authors:** Niklas Joisten, Alexander Schenk, Philipp Zimmer

**Affiliations:** ^1^Department of Performance and Health (Sports Medicine), Institute of Sport and Sport Science, Technical University Dortmund, Dortmund, Germany; ^2^Department for Molecular and Cellular Sports Medicine, Institute of Cardiovascular Research and Sports Medicine, German Sport University, Cologne, Germany

**Keywords:** exercise, animal model, metabolism, molecular mechanism, health

## Introduction

The beneficial multilevel effects of physical activity and exercise in the prevention and rehabilitation of various diseases are well-described (Pedersen and Saltin, 2015). In contrast, physical inactivity has been associated with the incidence of several chronic diseases, hospitalization, and mortality (Biswas et al., 2015). Knowledge about underlying mechanisms of the beneficial effects of physical activity and exercise on the one hand and the detrimental consequences of physical inactivity on the other hand is sparse. However, a better understanding of these mechanisms would help to improve activity recommendations and individualize exercise programs. Research efforts in humans primarily investigate molecular mechanisms in skeletal muscle, blood, and adipose tissue but are strongly limited regarding the number of measurements, passive control groups (due to ethical reasons), and availability of other organs/tissues.

In contrast, animal research provides access to organs such as the liver or the central nervous system, thereby enabling the opportunity to conduct in-depth analysis considering interorgan cross-talk under highly standardized conditions. Moreover, knockout and reporter animal models enable the investigation of the importance of specific genes or gene sets for the adaptation to exercise stimuli.

During the past decade, a rising number of promising articles (e.g., Agudelo et al., 2014; Pedersen et al., 2016; Takahashi et al., 2019; Brett et al., 2020; MacDonald et al., 2020) uncovered different metabolic mechanisms of exercise in animal models, which are either involved in the alleviation of disease progression (e.g., depression, hyperglycemia, and tumor growth) or reveal novel aspects of metabolism. However, it remains unclear whether these findings are only driven by “exercise-induced” effects or biased by levels of inactivity as they probably occur through housing conditions.

## Impairment of Habitual Movement Behavior in Studies Performed in Rodents

Most animal models investigating the impact of exercise on a specific aspect of metabolism or disease, compare at least one intervention group vs. a control group. Intervention groups usually receive a distinct amount of varying physical activity or exercise modalities over several weeks. On the contrary, control groups do not receive any intervention. Animals of both groups are typically housed in cages with controlled conditions as for example cage sizes, light/dark cycles, or nutrition. Existing guidelines and recommendations on laboratory animal science ensure adequate rodent housing conditions (Guillen, 2012). The cages should be large enough to allow the animals to naturally move, stand up and climb. Moreover, the aspiration of environmental enrichment aims to provide rodents the opportunity to express their natural behavior (e.g., paper tunnels, appropriate floors with adequate depth of specific material to allow hiding or digging behavior; Hutchinson et al., 2005). Despite these housing conditions, animals of control and intervention groups barely have the opportunity for habitual voluntary movement. Although knowledge about physical activity behavior of free-living mice is sparse, factors such as food, water, and territorial defending do not demand movement in captive mice used in animal trials (Latham and Mason, 2004). A recent investigation revealed that preventing climbing and reducing cage sizes in mouse studies impact outcomes that are associated with physical inactivity, such as bodyweight and fat mass. These results underline the influence of housing conditions on movement behavior (Roemers et al., 2019).

Against this backdrop, control groups, in particular, might be highly inactive in most exercise studies performed in rodents, thereby representing an inadequate control group paradigm to investigate increased physical activity or exercise-induced effects. The applied exercise volume in the intervention group may also need to be questioned since the common habitual level of physical activity is missing during day and night as well.

## Discussion

**Figure 1:**
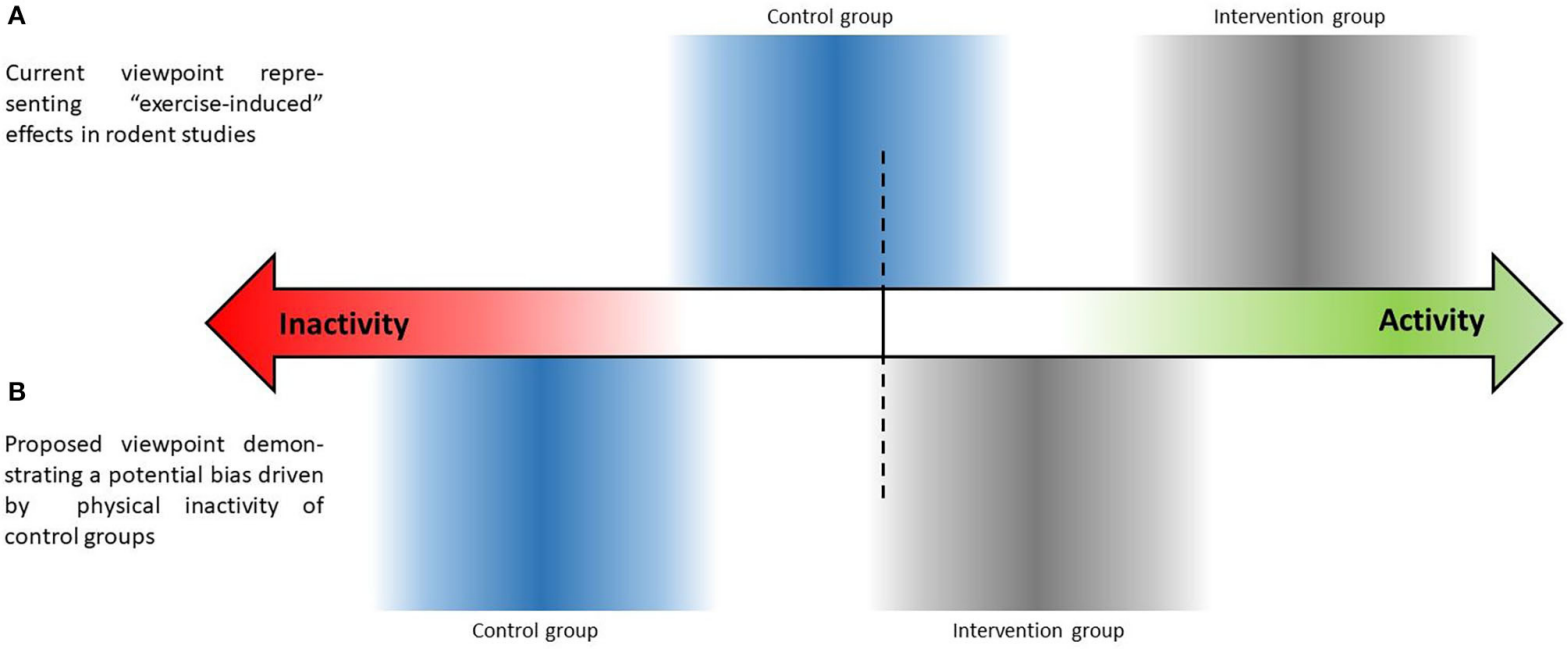
Schematic illustration highlighting a potential bias through (in)activity behavior during the intervention period in controlled exercise training studies performed in rodents.

As a consequence, the question arises whether animal studies with passive control groups really investigate “activity” - or “exercise-induced” effects - or rather the effects of physical “inactivity” (see Figure 1)? The possible inactivity of control groups may contribute to the failure of transferring the promising findings from animal studies to clinical trials, which is obviously also due to other reasons such as general differences between human and rodent biology (see e.g., Mestas and Hughes, 2004) or differences in the applied exercise training modalities (frequency, intensity, type, time). Indeed, people living in first world countries are often highly inactive as well, leading to an increased risk of developing various different chronic diseases. However, clinical trials define precise inclusion criteria that, for example, comprise daily physical activity behavior when applying exercise as an intervention, and further describe the investigated population, e.g., as “sedentary” (Arikawa et al., 2010; Cox et al., 2017; Amaro-Gahete et al., 2020). Clinical trials also have started to assess participants' activity behavior during the intervention across all study arms in order to check potential manipulation through changes in leisure time physical activity (Proschinger et al., 2019). In contrast, many exercise studies performed in rodents use highly standardized conditions with strong internal validity for the investigation of different aspects of metabolism or health, but do not provide any information on activity levels of the control and intervention groups beside the exercise intervention itself. If both intervention and control groups in rodents show impaired activity levels during the intervention period, the “exercise-induced” effects potentially solely alleviate mechanisms of degeneration.

With this opinion article, we do not seek to provide *evidence* for the inactivity of control groups of exercise studies performed in rodents. We seek to highlight the urgent need of accurate activity controlling within each exercise study performed in rodents to gain important information on the movement behavior of rodents during the intervention period. Both, the inactivity of all animals regardless group allocation as well as randomly occurring differences in activity levels between control and intervention groups could possibly bias the study results. Furthermore, researchers might consider providing voluntary wheel running in the control group as an opportunity to approach habitual movement behavior. Consequently, intervention groups would need further doses of exercise in addition to their voluntary amount of wheel running. The definition of specific activity doses for control groups (e.g., aerobic exercise twice per week) would be another opportunity to avoid inactivity. This more standardized strategy of inactivity compensation might also prevent activity levels comparable to long-term exercise training programs caused by the unlimited access to a running wheel during the whole intervention period. However, it remains questionable whether access to a running wheel for example twice per week is feasible when the mice find it repeatedly locked for the remaining days. Additionally, wheel running may not induce similar benefits as natural movement behavior and leads to increased data variability, possibly representing a different source of bias in exercise studies performed in rodents. Natural data variability regarding activity and other variables is observed in human (exercise) research as well but should not necessarily be considered as bias. Natural data variability for example regarding activity levels might also aid to bridge the gap between basic exercise research in rodent and clinical exercise trials. Certainly, larger sample sizes are required to reveal statistical effects in case of higher data variability. In clinical research, a priori power calculations attempt to prospectively estimate a sufficient sample size to capture statistical effects. Although the variability cannot be estimated a priori, this approach might help researchers conducting animal trials that want to converge basic animal and human research to facilitate study results transferability.

Due to the discussed methodological issues and the fact that it remains unclear which amount of activity is needed to compensate inactivity due to housing conditions, a deeper understanding of (in)activity levels in rodent exercise studies is needed at first. We encourage future research approaches to investigate voluntary activity levels in large and environmentally enriched cages compared to standard housing conditions. Based on the assessment of “norm” activity values, adequate interventions to compensate inactivity can be developed and/or housing conditions might be adapted.

Nevertheless, accurate activity controlling in exercise studies performed in rodents represents the first step to screen for the possible confounding effect of (in)activity. Several analysis systems to assess distinct behavioral parameters including activity in various housing conditions have been reviewed elsewhere (Bains et al., 2018). Using these systems in the context of rodent exercise studies will provide a better understanding of the role of housing conditions on inactivity and its potential effects on the respective outcomes of interest.

In conclusion, we believe that there is a need for accurate activity controlling in both intervention and control groups in rodent exercise trials. To increase our understanding of how promising results from exercise trials performed in rodents are achieved, (in)activity of the rodents should be considered as potential confounding factor in all study arms.

## Author Contributions

NJ drafted the manuscript. AS and PZ revised it critically for important intellectual content. All authors contributed to the conception and approved the final version of the manuscript.

## Conflict of Interest

The authors declare that the research was conducted in the absence of any commercial or financial relationships that could be construed as a potential conflict of interest.
